# Circulating LH/hCG receptor (LHCGR) may identify pre-treatment IVF patients at risk of OHSS and poor implantation

**DOI:** 10.1186/1477-7827-9-161

**Published:** 2011-12-23

**Authors:** Anne E Chambers, Krishnaveni P Nayini, Walter E Mills, Gillian M Lockwood, Subhasis Banerjee

**Affiliations:** 1 Department of Clinical Biochemistry, Heartlands Hospital, Birmingham B9 5SS, UK; 2 Midland Fertility Services, Aldridge WS9 8LT, UK; 3 Origin Biomarkers, BioPark, Broadwater Road, Welwyn Garden City, Hertfordshire, AL7 3AX, UK

**Keywords:** soluble LHCGR, LH-LHCGR, AMH, FSH, OHSS, IVF, Pregnancy

## Abstract

**Background:**

Successful pregnancy via *in vitro *fertilization (IVF) depends on the recovery of an adequate number of healthy oocytes and on blastocyst implantation following uterine transfer. Two hormones, LH and hCG, utilize a common LH/hCG receptor (LHCGR), variations in which have profound implications in human reproduction. Soluble LHCGR (sLHCGR) is released from experimental cell lines and placental explants and it can be detected in the follicular fluid and serum.

**Methods:**

To evaluate the impact of circulating soluble LHCGR (sLHCGR) in fertility treatment, we measured sLHCGR and LH-sLHCGR complex in serum from women seeking IVF using specifically developed quantitative enzyme-linked immunosorbent assays (ELISA). Following an IVF cycle of treatment, patients were grouped according to oocyte yield into low (lower than or equal to 7 oocytes), intermediate (8-14 oocytes) and high (greater than or equal to 15 oocytes) responders and pregnancy outcome noted.

**Results:**

Pre-treatment sLHCGR identified many women at risk of ovarian hyperstimulation. Low levels of sLHCGR were associated with pregnancy in both high and low responders but sLHCGR did not significantly affect the treatment outcome of intermediate responders. Low responders who failed to become pregnant had high levels of circulating sLHCGR bound to LH (LH-sLHCGR).

**Conclusions:**

Pre-treatment measurement of sLHCGR could be used to tailor individual fertility treatment programs and improve outcomes by avoiding ovarian hyperstimulation and poor embryo implantation.

## Background

Given that about three-quarters of human embryos created by assisted reproductive technology (ART) such as IVF and ICSI fail to produce live births following uterine transfer [1], there has been increasing interest in identifying pre-treatment factors that may indicate the likelihood of success with fertility treatments. The follicle stimulating hormone [FSH] and luteinizing hormone [LH], play central roles in the maturation of the ovarian follicles and ovulation. Following ovulation, the remaining cells of the follicle are luteinized by LH and secrete progesterone plus oestradiol which are necessary for implantation of the embryo [2]. One of the important aspects of the female reproductive cycle is that ovulation is controlled by a pulsatile secretion of LH (LH surge) that is subject to individual variation [3]. In controlled ovarian stimulation (COS), this variability of ovarian activities is temporarily eliminated by GnRH agonists/antagonists, while at the same time the ovarian functions are regulated by exogenously injected FSH and LH/hCG [4-7].

In addition to routine estimation of LH and FSH, the serum concentrations of anti-mullerian hormone (AMH) are often measured prior to COS. While LH or FSH alone or together provide a measure of the ovarian activity, AMH is the reliable marker for functional ovarian reserve [8]. Moreover, high AMH levels (> 45 pmol/L) are indicative of polycystic ovary (PCO) and these patients are usually susceptible to ovarian hyperstimulation syndrome (OHSS) following hormonal induction. The serum AMH concentrations, being independent of the phase of the reproductive cycle, also appear to have some predictive value with respect to the pregnancy outcome [9,10].

The receptors expressed on the surface of target cells for LH and human chorionic gonadotrophin (hCG) binding and signalling are identical and are therefore described as the LH/hCG receptor or LHCGR [11]. In addition to ovary and testis, various isoforms of LHCGR are expressed almost ubiquitously in primary and secondary reproductive organs (uterus, fallopian tube, placenta and breast) and in a variety of extragonadal tissues including vascular endothelial and smooth muscle cells, brain, lymphocytes, macrophages, skin as well as foetal tissues [12,13]. The physiological significance of the extragonadal expression of LHCGR remains unexplained.

The earliest experimental evidence of the cell-free existence of LH receptor was its hCG-affinity purification from porcine follicular fluid [14]. The detection of a M_r _80-90K LH-LH receptor complex from Leydig cell culture media [15] and a M_r _30-60K LH-receptor binding protein in serum from uremic boys suffering from hypogonadism provided further clues to the extracellular appearance of soluble LH receptor. Notably, both soluble LH receptor and the hCG-receptor complexes have been shown to be released from cells transfected with cloned LH receptor [16-18]. However, the clinical significance of the cell-free soluble LHCGR remained unknown due to the lack of a convenient experimental system for quantitative estimation of the soluble receptor in body fluids.

We have recently shown that soluble LH/hCG receptor (sLHCGR) is released from LHCGR-transfected cells, placental explants [19] and into the bloodstream of pregnant women. Here, we describe the development and use of quantitative ELISA assays for the measurement of sLHCGR and hormone-receptor complex (LH-sLHCGR) in the serum of women presenting for IVF treatment. The assays described detect the analytes in follicular fluid, serum and plasma samples from the same patient. Our results demonstrate that women with high pre-treatment serum sLHCGR or LH-sLHCGR are usually low responders, produce reduced number of oocytes (≤ 5 oocytes) and are less likely to become pregnant. In addition, low to undetectable pre-treatment circulating LHCGR/LH-receptor complex is associated with high ovarian response (≥ 15 oocytes) and OHSS. The clinical applications of these assays in predicting ovarian response to fertility treatment and improving implantation potential following embryo transfer are discussed.

## Methods

### Antibodies

Purified LHR29 monoclonal antibody [19,20] was initially provided by Dr Hugues Loosfelt (INSERM, France) and subsequently the antibody producing clone was obtained from ATCC (Clone ID CRL-2685). The epitope for the LHR29 monoclonal antibody was mapped using LHCGR recombinant proteins expressed in CHO cells [19]. It resides within amino acid residues 229 to 291 of the N-terminus extracellular domain (ECD) of LHCGR. A 19 amino acid residue LHCGR peptide (LHCGR 209-227; Swissprot: locus LSHR_HUMAN, accession P22888) was used to produce a second monoclonal antibody, clone 5A10C9. This antibody was purified from culture supernatant by Protein A affinity chromatography. The monoclonal antibody against luteinizing hormone β (LH β) clone 2LH2-L1 was obtained from HyTest Ltd. (Turku, Finland). In addition to this, a variety of polyclonal antibodies against LHCGR and monoclonal antibodies against LH β were tested during assay development. Antibodies were conjugated to Horse Radish Peroxidase using a Lightning-Link HRP conjugation kit according to the manufacturer's instructions (Innova Biosciences, Cambridge, UK). Briefly, 10 μL of LL-modifier was added to 100 μg (100 μL) of affinity purified antibody and was conjugated either for three hours or overnight before the reaction was stopped using 10 μL of LL-Quencher reagent for 30 minutes. Conjugated antibodies were stabilized by the addition of 10 μL Peroxidase Stabilizer (Cosmo Bio Co. Ltd., Japan) and stored at 4°C for up to six months. The specificity of the assay was established by a variety of controls including mock/LHCGR ECD-expressing Chinese Hamster Ovary (CHO) cell extracts and isotype-specific IgGs from rabbit, mouse and goat.

### ELISA protein standards

The expression of soluble LHCGR (sLHCGR, N-terminal 336 residues of the ECD) as a thioredoxin fusion protein in *Escherichia coli *(*E*. *coli*) bacteria carrying mutations in both thioredoxin reductase (*TrxB*) and glutathione reductase (*gor*) genes has been reported [21]. This chimeric sLHCGR was shown to have a similar specificity and affinity for hCG as the intact native LHCGR [21]. This protocol was therefore followed in order to produce sLHCGR standard for ELISA assays. Briefly, cDNA encoding the N-terminal 316 amino acid residues of the LHCGR ECD was cloned into the Eco RI (5') and Sal I (3') sites of pET32a(+) vector (Novagen, USA) to generate an *E. coli *bacterial clone. With respect to orientation, the clone contained two histidine repeat tags, one at the C-terminus and the other sandwiched between the LHCGR ECD and TrxB at the N-terminus. The DNA sequence of the insert was confirmed prior to transformation of *E*. *coli *(Origami strain *trxB gor *(DE3) pLysS (kanR, tetR, CmR), Novagen, USA). The protocol for expression of the LHCGR fusion protein and affinity purification through Ni-NTA resin column (Qiagen) were exactly as described [21]. The estimated relative molecular mass of the fused sLHCGR was 57541 with pI of 6.15. The affinity purified protein was > 90% pure and the yield varied from 6.63 to 7.34 mg/L. The affinity purified protein was stored at a concentration of 0.255 mg/mL (4.4316 μM) in 25% glycerol at -20°C.

Unlike sLHCGR standard, the creation of an *in vitro *LH-LHCGR complex standard was less straight forward. Previous work [22] had established that a tethered single chain hCG-LHCGR cloned in baculovirus and expressed in insect cells was functional with respect to ligand-receptor interaction. Moreover, this yoked hCG-LHCGR ECD was shown to be secreted from insect cells at levels 20-fold higher than conventional eukaryotic expression systems [22]. Our goal was to produce a yoked LH β-sLHCGR single chain protein containing the epitopes recognized by both LH β and LHCGR antibodies. Therefore, the open reading frame encoding the entire 141 amino acids of hLH β was synthesized (Source: UniProtKB/Swiss-Prot;Acc:P01229). A linker sequence encoding the hCG β C-terminal peptide (CTP, constituting amino acids 116-145 of hCG β) was ligated at the 3' end of the above construct (hLH β-CTP). The addition of the CTP sequence has been shown to stabilize the expressed fusion protein [22]. A cDNA clone encoding 115 to 291 amino acid residues of the N-terminal end of LHCGR ECD was produced. The hLH β-CTP was ligated to the 5'-end of the modified LHCGR and cloned into p3XFLAG-CMV-14 vector to create an *E. coli *bacterial clone. The hLH β-sLHCGR complex was first expressed in transfected CHO cells and the specificity of the yoked hLH β-sLHCGR protein was established by testing anti-LHCGR, anti-hLH β and anti-FLAG monoclonal antibody binding of the recombinant and mock transfected CHO extracts in plate assays. For large scale production, the protein was expressed in baculovirus transfected sf9 insect cells in order to ensure correct eukaryotic glycosylation of the LH moiety of the fusion protein. Purification was achieved by loading the culture supernatant onto FLAG M2 affinity gel, followed by elution with TBS (50 mM Tris-HCl, 150 mM NaCl, pH 7.4) containing 200 ng/μL FLAG peptide (synthetic octapeptide, N-Asp-Tyr-Lys-Asp-Asp-Asp-Asp-Lys-C molecular weight 1013.0). The yield of the yoked hLH β-sLHCGR protein was 0.43 mg/L with an estimated purity of 50%. The relative molecular mass of the fusion protein backbone was 40850 (pI 6.65). The molecular mass of glycosylation was not included in quantification calculations. The affinity purified protein was stored at a concentration of 52.5 μg/mL (1.2851 μM) in 25% glycerol at -20°C.

ELISA standards were two-fold serially diluted with extensive mixing through 25 mM Bicine (Fluka), 50 mM Tris pH 7.8, 170 mM NaCl in uncoated vinyl mixing plates (Costar, UK) immediately prior to loading onto a pre-coated ELISA plate. The concentration of the first dilution for each standard was as follows: sLHCGR, 39.88 picomoles per mL; LH-sLHCGR, 20.5616 picomoles per mL.

### ELISA procedure

Standard ELISA methods were followed for quantitative analysis of the sLHCGR and LH-LHCGR protein in the serum samples. Details for antibody coating of ninety-six-well plates, antigen binding, antigen detection and data analysis will be published elsewhere (manuscript in preparation).

### Human sera and sLHCGR and LH-sLHCGR protein standards show dilution dependent effects in ELISA

**Figure 1:**
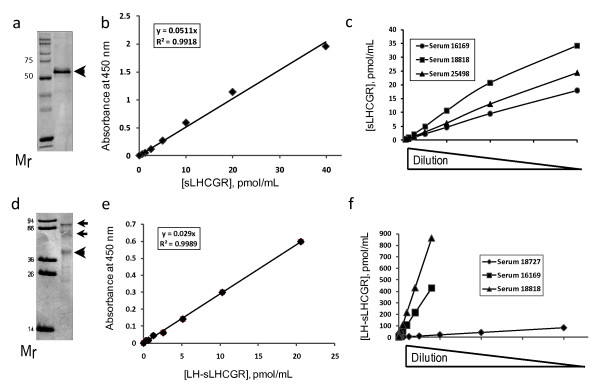
**The sensitivity of sLHCGR and LH-sLHCGR ELISAs**. The purity of the affinity purified recombinant LHCGR protein extracellular domain (a, indicated by an arrow) and LH-sLHCGR (d, indicated by arrows) proteins were examined in SDS-polyacrylamide gels-stained with coomassie blue. The small arrows above the major band in d) are either glycosylated or dimers because these bands react with anti-FLAG and LHR29 monoclonal antibodies. The sensitivity of the ELISAs was verified by linear responses to the dilution effect of sLHCGR (b) and LH-sLHCGR protein calibrators (e). The sensitivities of these assays were further examined by assaying serially diluted serum samples from three patients with known concentration of sLHCGR (c) and LH-LHCGR (f). Each data point in b and e represents mean value obtained from six independent dilution of the standards.

The specificity of the capture-detection system for ELISA was first established employing anti-FLAG affinity purified recombinant LHCGR proteins (LHCGR ECD, amino acid residues 1-291) expressed in CHO cells [19]. The estimated purity of this affinity purified LHCGR standard from CHO cell extracts was 40-50%, as judged by SDS-PAGE and western blots. Subsequently, bacterially expressed affinity purified recombinant LHCGR ECD which had a consistent purity of > 90% (see Methods) was used for all sera analyses (Figure 1a). This sLHCGR standard produced a linear dilution dependent response when LHR29 and 5A10C9 were used as capture and detection antibodies, respectively. The sensitivity (limit of detection) of the assay was less than 0.62 pmol/mL (Figure 1b). The dilution dependent linear response of the sLHCGR ELISA system was further established by examining the dilution effect of three early pregnancy sera (Figure 1c). The LH-sLHCGR standard, created by tethering LH to a portion of the LHCGR ECD was expressed in insect cells to preserve glycosylation and the anti-FLAG affinity purified fusion protein was approximately 50% pure (see Methods, Figure 1d). Serially diluted LH-sLHCGR protein was captured by 5A10C9 and was detected by HRP-conjugated anti-LHβ monoclonal antibody. Based on the estimated purity of the standard, the sensitivity (limit of detection) of the assay was < 0.64 pmol/mL (Figure 1e). Although the affinity purified LH-sLHCGR standard appeared as three bands on western blot (Figure 1d), it was unclear whether all three were active on ELISA and whether they might be multimers of the protein standard, therefore purity calculations were based solely on the band of expected relative molecular mass (40850). In addition, the contribution of glycosylation to molecular mass was not included in molarity calculations. All measurements for LH-sLHCGR complex are therefore conservative, but consistent for inter-comparison of sera values. The dilution dependent linear response of the LH-sLHCGR ELISA system was further established by examining the dilution effect of sera from three female patients seeking fertility treatment (Figure 1f).

### Patients

This prospective study had a broad aim to examine the association of pre-treatment serum sLHCGR and LH-sLHCGR concentrations with treatment outcome for patients seeking fertility treatment at Midland Fertility Services (MFS), Aldridge, UK. This study was approved by West Midlands Research Ethics Committee, UK. Written informed consent was obtained from the participants of this study, with the understanding that consent could be withdrawn by the patient at any time without prejudice.

### Statistical analysis

The means, standard deviations, variance (anova), confidence intervals (CI) for each data set were computed using Analysis ToolPak (ATP) software. The significance of differences between groups was evaluated using two-sample paired t-test. ELISA assay standard curves were generated following examination of the natural log plot of the optical density (OD) at 450-620 nm for all the standard points to ensure that the points form a straight line.

The graph plotting and statistical analyses were carried out using the R statistical environment and the ggplot2 package running on a Linux operating system. Correlation testing was performed using the Pearson product moment method which is available in the standard R package. General file manipulations and data cleaning were implemented using the Awk programming language or custom programs written in Python. All software was open source and is freely available from publicly accessible sites.

## Results

### Clinical samples; regimens, oocyte yields, pregnancy outcomes and data collection

Baseline serum samples (cycle day 2-5) were obtained from 276 women who attended the clinic. Of these, 161 patients enrolled in an IVF or ICSI/IVF cycle. All samples irrespective of fertility treatment, were assayed for FSH, LH, AMH, sLHCGR and LH-sLHCGR; however, AMH together with LH data were not available for seven patients.

One mL follicular fluid samples were obtained during oocyte collection and were stored exactly as serum samples. For patients undergoing fertility treatment, the hormone data (FSH, LH, AMH) in conjunction with information on age, antral follicle count (AFC) and Body Mass Index (BMI) were used to determine the optimum dose for ovarian stimulation assuming a desired oocyte yield of 8-14 mature oocytes. Information on sLHCGR or LH-sLHCGR serum concentration, where known, was not used to alter patient treatment regimens. The majority of patients undertook a 'long-protocol' stimulation regimen with GnRH agonist down-regulation commenced in the luteal phase. Patients with a high baseline FSH level (> 10 IU/L) undertook a 'short protocol' stimulation regimen with GnRH antagonist control. Despite the attempt to 'normalise' the response in terms of oocyte yield, there was a wide variation with respect to the oocyte yield (range 1-39) and as expected there was a strong positive correlation between oocyte yield and AMH levels (see below). Given that there are no absolute cut-off values for 'low" and 'high responders' the patients were divided into three groups: High responders, with ≥ 15 oocytes; intermediate responders with 8-14 oocytes and low responders with a yield of ≤7 oocytes; 48.6 to 70.4% of the oocytes were fertilized; > 95% of the patients had two fresh embryos transferred. Each group was further sub-divided by the pregnancy outcome (*i.e*. pregnant or non-pregnant). In the high response group (54 patients), 17 (31.5%) became pregnant, 14 did not become pregnant and 23 (42.6%) had all their embryos frozen due to a significant risk of OHSS. In the intermediate responder group (54 patients), 27 (50%) became pregnant. In the low response group (53 patients), 13 (24.5%) became pregnant and 40 did not. Pregnancy was initially confirmed by hCG test and by ultrasound scan showing fetus at around 7 wk following transfer.

### sLHCGR is detected in follicular fluid, plasma and serum

In a comparative analysis, 8 pairs of pre-treatment serum and follicular fluid (following LH or hCG ovarian stimulation) from 8 patients undergoing fertility treatment were assayed for sLHCGR. Although the mean concentration of sLHCGR was always higher (≥1.9-fold) in the serum compared to the follicular fluid, concentrations in each followed a similar pattern. In some patients sLHCGR was undetectable in both serum and follicular fluid (data not shown). Concentrations of sLHCGR in paired plasma and serum samples from either pregnant or non-pregnant women were generally comparable (data not shown).

### Low and high serum AMH are inversely related to pre-treatment LHCGR/LH-LHCGR concentrations

**Figure 2:**
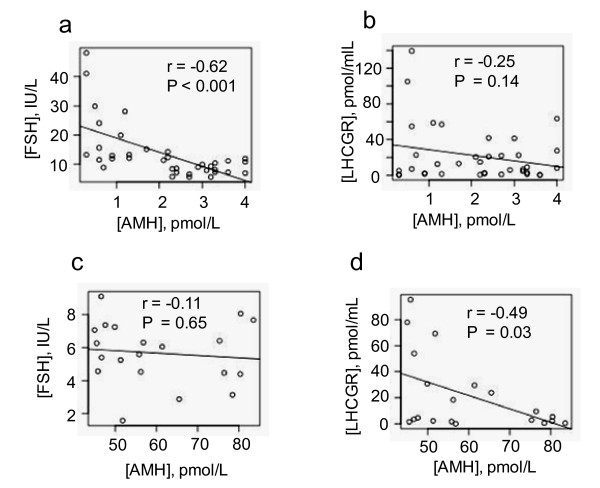
**High serum FSH and low sLHCGR are inversely related to the pre-treatment serum AMH concentrations**. The basal AMH concentrations at ≤ 4 pmol/L and ≥ 45 pmol/L were compared with corresponding FSH (a & c) and sLHCGR (b & d) concentrations. The correlation coefficient (*r*) and the level of significance (*P *value) are shown in each case.

We examined the relative concentrations of serum FSH, sLHCGR and LH-sLHCGR at very low and high AMH levels. As expected, at very low AMH (0.3-2.2 pmol/L), the FSH concentration was significantly high (*P *< 0.001) with a correlation coefficient of *r *= -0.62 (Figure 2a). Moreover, at these levels of AMH (≤ 4 pmol/L), the average FSH:LH ratio was 2.3:1. At low AMH (≤ 4 pmol/L), the sLHCGR concentrations were relatively high (> 20 pmol/mL) in 35.1% (^13^/_37_) of patients (Figure 2b) with correlation of *r *= -0.25 (*P *= 0.14). Serum sLHCGR was similarly inversely related (*r *= -0.49, *P *= 0.03) at AMH range of ≥45 pmol/L (Figure 2d). In ~70% (^16^/_23_) of these women, the serum LH-sLHCGR was either undetectable (52.2%) or ≤1.0 pmol/mL (17.4%, see below). Moreover, unlike the low AMH group (≤ 4 pmol/L), FSH failed to significantly correlate with AMH (Figure 2c) and the FSH:LH ratio in patients with high AMH (≥45 pmol/L) was 1.2:1 (data not shown). Together, these results suggest that low FSH:LH ratio (≤1:1) and undetectable or low sLHCGR/LH-sLHCGR could be independent indicators of PCO/OHSS.

### More than 30% of all patients tested have high serum sLHCGR

**Figure 3:**
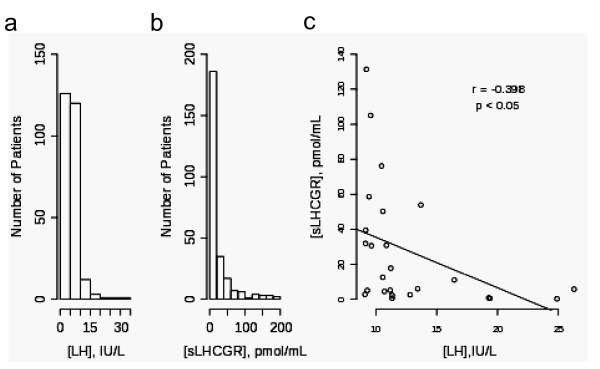
**A large proportion of women (> 30%) seeking fertility treatment have > 20 pmol/mL, high pre-treatment sLHCGR**. The frequency and distribution of the concentrations of a) LH and b) sLHCGR measured in 274 women seeking fertility treatment are shown. Additionally, the LH concentrations ≥ 9.1 IU/L are inversely related to the sLHCGR levels.

To establish the prevalence and concentration range of sLHCGR in women presenting for IVF treatment, early cycle LH and sLHCGR levels were measured and compared in 273 patients. The LH concentrations in 47% (128 patients) and 46% (126 patients) of the samples were 0.07-5.0 IU/L and 5.0-10.0 IU/L, respectively, while the remaining 7% (19 patients) samples had > 10.0 IU/L (Figure 3a). When compared, 52% (141 patients) and 16% (45 patients) of the samples had serum sLHCGR of 0-9.9 pmol/mL and 10-20 pmol/mL, respectively (Figure 3b). The remaining 32% (87 patients) had high concentrations of sLHCGR (> 20 pmol/mL). High levels of early cycle serum LH (≥10 IU/l) is usually associated with PCO and susceptibility to heightened ovarian response on controlled ovarian stimulation. Therefore, LH values > 9 IU/L were compared with corresponding sLHCGR levels. This analysis revealed that a negative correlation (*r *= -0.397, *P *< 0.05) exists between concentrations of the hormone and the soluble receptor (Figure 3c).

### Age negatively correlates with pre-treatment sLHCGR levels in non-OHSS women

**Figure 4:**
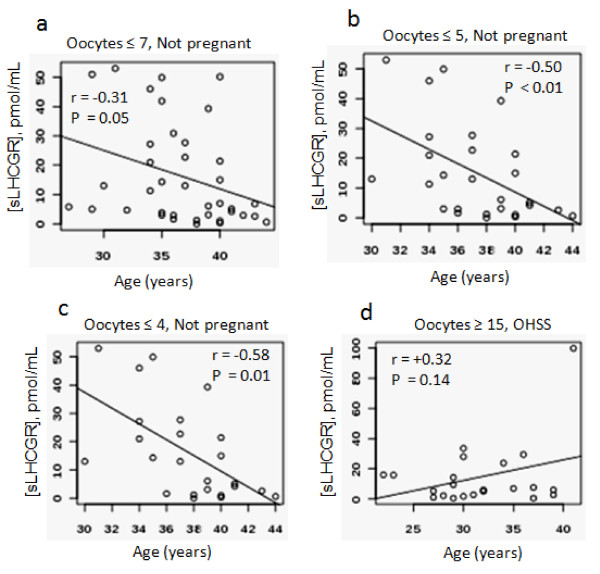
**Younger women among low responders to COS have higher serum sLHCGR**. Patients yielding a) ≤ 7, b) ≤ 5 and c) ≤ 4 oocytes with respect to their age were plotted. The correlation between sLHCGR and age among high responders (≥ 15 oocytes) with OHSS is shown (d). The correlation coefficient (*r*) and the level of significance (*P *value) are shown for each group.

Comparative analysis of AMH with age (range 21 to 47 yrs) on 269 women showed a correlation coefficient of *r *= -0.411, confirming that the women in the higher age group tend to have low AMH and reduced ovarian reserve (data not shown). Based on this observation, we asked whether pre-treatment sLHCGR correlates with patient age. In those that were low responders age had a significant impact on sLHCGR levels (Figure 4a-c). In responders yielding ≤7 oocytes age was inversely correlated with sLHCGR serum concentrations (*r *= -0.31, *P *= 0.05, Figure 4a). The age-effect became more evident among responders producing ≤ 5 (*r *= -0.50, *P *< 0.01) or ≤ 4 (*r *= -0.58, *P *= 0.01) oocytes (Figure 4b and 4c). This suggests that the younger women who exhibit a low response to COS would be more likely to have higher serum sLHCGR than older women with the same oocyte yield. Despite a lack of statistical significance (*P *= 0.14), it is interesting to note that an opposite correlation may exist in the case of high responders (oocytes ≥ 15) who exhibit OHSS; where age tended to be positively correlated (*r *= +0.38) with sLHCGR concentrations (Figure 4d). Although not statistically significant, these results may indicate that older women with high AFC might produce increased sLHCGR.

### High pre-treatment serum sLHCGR is negatively associated with oocyte yield

**Figure 5:**
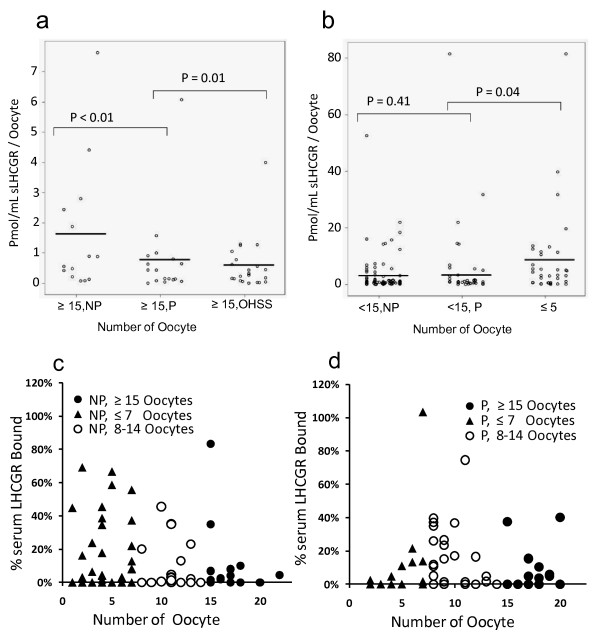
**Quantitative inhibition of the release of oocytes by high pre-treatment serum sLHCGR following COS**. The ratio, sLHCGR:Oocyte, was determined by calculating the sLHCGR molecules (pmol/mL) per oocyte in women who had ≥ 15 oocytes (a) and those who yielded b) < 15 and ≤ 5 oocytes. The percentage of sLHCGR bound to LH (sLHCGR/LH-sLHCGR%) with respect to the yield of oocytes and treatment outcome (c & d). The level of significance (*P *values) determined by comparing different groups are shown. NP, Not pregnant; P, Pregnant.

The relative yield of oocytes with respect to pre-treatment sLHCGR was analysed in high (≥ 15 oocytes) and low (< 15 oocytes) responders by estimating the mean pmol/mL sLHCGR per oocyte. In the high response group, women with OHSS had the lowest amount of mean sLHCGR/oocyte (0.58 pmol/mL), compared to non-pregnant (1.63 pmol/mL) and pregnant (0.77 pmol/mL) women (Figure 5a) Overall, for those who yielded > 15 oocytes, irrespective of pregnancy or OHSS, the average level of sLHCGR per oocyte was 0.99 pmol/mL. However, for those with 5-15 oocytes, both pregnant (5.7 pmol/mL) and non-pregnant women (5.1 pmol/mL), had an average pre-treatment sLHCGR concentration of 5.4 pmol/mL per oocyte (Figure 5b), which is over 5-fold more than those yielding 15 or more oocytes. A similar analysis of the low responders (oocytes ≤5) showed an even higher concentration of sLHCGR per oocyte (8.7 pmol/mL/oocyte, Figure 5b). These results may indicate that high pre-treatment sLHCGR serum concentrations could alter functional LH levels leading to an inhibition of the release of oocytes following COS.

### High LH-sLHCGR concentrations correspond with poor treatment outcomes in low responders

The results shown above (Figure 5a and 5b) prompted examination of the fraction of sLHCGR bound to the hormone (LH-sLHCGR). The percentage of sLHCGR bound to LH (LH-sLHCGR) with respect to the yield of oocytes was analyzed for women who did not become pregnant (Figure 5c) or who became pregnant (Figure 5d). Of those who produced few oocytes (≤7) and who failed to become pregnant, many had a larger fraction of the serum sLHCGR bound to LH. Indeed, the saturation levels of sLHCGR with LH (expressed as a percentage of sLHCGR/LH-sLHCGR) in those who produced few oocytes (≤7) and did not become pregnant were on average similar to those with an intermediate number of oocytes (8-14 oocytes) who did become pregnant (15% versus 14% respectively). Intriguingly, in those with an intermediate number of oocytes (8-14 oocytes) who failed to become pregnant, the average percentage saturation of sLHCGR by LH was just 7%. By contrast, the average percentage saturation of sLHCGR by LH in 12 of 13 women who produced few oocytes (≤7) and who did become pregnant was 6%, suggesting that a downward shift in sLHCGR saturation levels may have enabled pregnancy in these low responders. A similar shift in sLHCGR saturation levels was noted in high responders, where those who became pregnant had a lower saturation level (7%) compared to those who failed to become pregnant (11%). Overall, these data suggest that the saturation level of sLHCGR by LH may be an important pregnancy determinant. High pre-treatment LH saturation levels of sLHCGR, coupled with either a low or high oocyte yield following COS, may be a useful indicator of poor treatment outcome.

### Low serum sLHCGR/LH-sLHCGR favours embryo implantation in high and low responders

**Figure 6:**
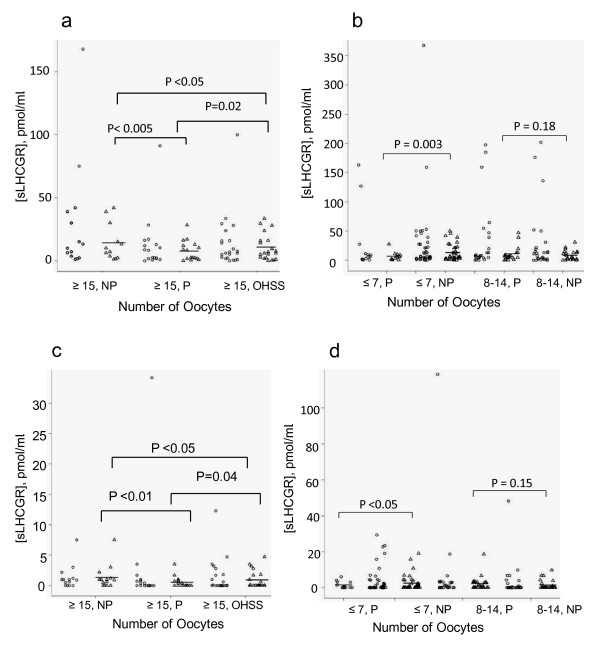
**High pre-treatment serum sLHCGR and LH-sLHCGR reduce embryo implantation**. The serum sLHCGR (a & b) and LH-sLHCGR (c & d) concentrations were plotted for patients yielding ≥ 15 oocytes (a & c) and < 15 oocytes (b & d). Each data set was plotted in duplicate (open circle and triangle), where extremely high values (open circle) in each data set were excluded (open triangle) in order to compare the data representing > 90% of the patients. The cut-off values for a) and b) were 50 and 100 pmol/mL, respectively and for c) and d) were 10 and 20 pmol/mL, respectively. The bar indicates the mean value for each condition below the cut-off values. The level of significance (*P *values) determined by comparing different groups are shown. NP, Not pregnant; P, Pregnant.

sLHCGR/LH-sLHCGR data for each clinical condition (high, intermediate and low response) in the context of each outcome (with pregnancy, without pregnancy or OHSS) are presented in dot-plots (Figure 6) and summarized in Table 1. Each condition has been represented by two sets of data: with and without cut-off values (open circle and triangle, respectively). To obtain more accurate estimates reflecting > 90% of the population, outliers were not incorporated in statistical analysis (Figure 6). The mean pre-treatment serum sLHCGR and LH-sLHCGR levels in high (oocytes ≥ 15), intermediate (oocytes, 8-14)) and low responders (oocytes ≤7) are shown in Figure 6 and in Table 1. In high responders (oocytes ≥ 15), mean sLHCGR (Figure 6a) and LH-sLHCGR (Figure 6c) levels were significantly lower than in women who failed to become pregnant. Similarly, women with OHSS had lower sLHCGR/LH-sLHCGR than the corresponding non-pregnant women (Figure 6a &6c). Among low responders (oocytes ≤7), high sLHCGR/LH-sLHCGR is also significantly associated with a lack of embryo implantation. Levels of sLHCGR/LH-sLHCGR in women with intermediate response (oocytes, 8-14) had little effect on pregnancy outcome (Figure 6b &6d). However, saturation levels of sLHCGR differed between those who achieved pregnancy and those who did not (Figure 4c and 4d). Together, these results suggest that while high serum LHCGR/LH-LHCGR inhibit embryo implantation in high (oocytes ≥ 15) and low responders (oocytes ≤7), women yielding optimum number of oocytes (8-14) are unaffected by their absolute pre-treatment sLHCGR concentrations. As shown in Table 1, women with highest yield of average number of oocytes experiencing OHSS (FAE), had highest serum AMH and lowest mean FSH:LH ratio (0.9 ± 0.3). Moreover, low responders (oocytes ≤7) had highest FSH:LH ratio (1.9 ± 1.4).

**Table 1:** A summary of the mean serum hormone concentrations (AMH, LH and FSH, FSH:LH ratio), receptor (sLHCGR) and LH-LHCGR concentrations and the number of oocytes with respect to the fertility treatment outcome

Hormones Receptors	Oocytes ≥ 15 Not Pregnant N = 13	Oocytes ≥ 15 Pregnant N = 16	Oocytes ≥ 15 OHSS; FAE N = 22	Oocytes ≤ 7 Not Pregnant N = 38	Oocytes ≤ 7 Pregnant N = 11	Oocytes 8-14 Not Pregnant N = 23	Oocytes 8-14 Pregnant N = 25
Number of oocytes	16.9(± 1.1)	17.1(± 0.8)	24.4 (± 0.3)	4.2 (± 0.6)	5.0 (± 1.0)	10.9 (± 0.6)	9.8 (± 0.7)
AMH, pmol/L	19.3(± 5.2)	18.3(± 4.1)	46.6 (± 9.9)	10.1 (± 3.2)	5.6 (± 2.6)	13.3 (± 3.7)	12.9 (± 3.6)
sLHCGR, pmol/mL	14.3(± 7.7)	7.9 (± 3.8)	9.9 (± 4.2)	16.2 (± 6.7)	6.9 (± 4.6)	19.5 (± 17.9)	15.2 (± 7.5)
LH-LHCGR, pmol/mL	1.35(± 1.0)	0.53(± 0.4)	0.88 (± 0.6)	2.98 (± 2.1)	1.4 (± 1.0)	3.0 (± 3.67)	2.48 (± 1.6)
LH, IU/L	5.9 (± 1.0)	4.5 (± 0.9)	7.3 (± 2.7)	5.9 (± 1.2)	5.1 (± 1.1)	4.9 (± 0.7)	5.2 (± 0.8)
FSH, IU/L	6.5 (± 0.7)	6.7 (± 1.0)	6.3 (± 0.9)	9.0 (± 1.3)	9.7 (± 1.6)	7.0 (± 0.7)	7.7 (± 0.9)
FSH/LH	1.1 (± 0.7)	1.5 (± 1.1)	0.9 (± 0.3)	1.5 (± 1.0)	1.9 (± 1.4)	1.4 (± 1.0)	1.5 (± 1.1)

No Pregnancy, Pregnancy and OHSS shown in Figure 6. The numbers in parentheses are confidence intervals (± CI).

Of 23 patients with OHSS, one became pregnant spontaneously, two did not participate in further treatment and for one patient, frozen embryo transfer (FET) is scheduled in the near future. Of the remaining patients (nineteen), twelve failed to become pregnant and seven were pregnant following FET; five of the seven who became pregnant had pre-treatment sLHCGR below 10 pmol/mL (data not shown).

## Discussion

Here we describe the development and use of two ELISA assays that quantitatively measure sLHCGR and sLHCGR in complex with LH (LH-sLHCGR) in human serum from women seeking fertility treatment. In addition to serum, these ELISA assays could quantitatively measure sLHCGR and LH-sLHCGR in human plasma and follicular fluids.

Serum AMH is increasingly used in fertility treatment as an indicator of ovarian reserve. Very low (0-4 pmol/L) and very high (≥45 pmol/L) AMH concentrations are associated with reduced oocyte yields and PCO (risk of OHSS) following COS, respectively [8]. Our data demonstrate the expected correlation between AMH levels and oocyte yield in women who exhibited OHSS and had all embryos frozen. However, high AMH (> 39 pmol/L) was a positive predictor for only 52% (12/23) of these patients; the remaining 48% (11/23) had AMH values ranging from 7.6-35 pmol/L. Regarding pregnancy, like many other reports [23-26], we found no correlation between AMH and the embryo implantation potential. We have described three categories of patients seeking fertility treatment with respect to the basal (pre-treatment) serum concentrations of sLHCGR/LH-sLHCGR: undetectable to very low (low), intermediate (optimal) and very high. Patients who were high (oocytes ≥15) and low responders (oocytes ≤7), had fresh transfers and became pregnant had soluble receptor levels that were significantly lower than those who did not become pregnant. Comparing pregnancy rates in low and high responders we found that the levels of LH complexed with sLHCGR were low in those who became pregnant (low and high responders) and higher in those who did not become pregnant. Although embryos were not transferred, the OHSS group (oocytes ≥15) also had low sLHCGR/LH-sLHCGR and FSH:LH ratio. A comparison of pregnancy rates among those producing an intermediate number of oocytes (8-14) revealed the opposite; those who became pregnant had higher levels of LH complexed to sLHCGR than did those who did not become pregnant. The results for complexed LH-sLHCGR amongst intermediate responders were not considered statistically significant and would require a larger study to verify a significant difference. Nevertheless, we believe that this observation merits discussion because, if verified, it could indicate that an optimum saturation level of sLHCGR that correlates with oocyte yields, is important for pregnancy. Moreover, if verified, this result might suggest an important biological function for sLHCGR in modulating LH availability, *e.g*. by acting as an LH reservoir as observed for IL-4 and growth hormone and tumor necrosis factor receptors [27-30]

Given that the production of soluble polypeptide hormone and cytokine receptors is regulated by alternative splicing, protease activation, secretion of membrane vesicles [31], ligand-mediated receptor activation [15] and stress [19], the higher concentrations of the sLHCGR/LH-sLHCGR that we have observed could be attributed to infection, endometriosis, ovarian and adrenal pathology, obesity, insulin resistance and other metabolic diseases. Notably, a recent report suggests hCG stimulates LHCGR expression in lymphocyte during controlled ovarian stimulation and is linked to improved implantation [32] On the other hand, undetectable to low circulating sLHCGR/LH-sLHCGR might reflect reduced hormonal activation and down regulation of sLHCGR synthesis as observed in Down's syndrome placenta [33,34]. This may suggest that like other cytokine and hormone receptors [27-30], the production of sLHCGR is regulated.

What could be the patho-physiological significance of sLHCGR in the context of COS and implantation of the embryo? There are two possible mutually non-exclusive ways the sLHCGR could affect the LH/hCG functions: sLHCGR is intrinsically a specific serum binding protein [28-30] which stabilizes or prevents degradation of the LH/hCG until the hormone is delivered to the traditional membrane-bound receptor. In extreme conditions (low or undetectable sLHCGR), leading to OHSS and high response might reflect an unregulated burst of stimulation resulting from hormonal induction. This is consistent with fast and slow disappearance (double exponential curves) of LH and hCG reported a few decades ago [35]. Accordingly, very short serum half-life of variably glycosylated LH (20-80 min, [35,36]) compared to FSH (several hrs, [37]) and hCG (1-3 days, [38]) suggests that combined and sustained LH-FSH induction of ovarian functions would require receptor-mediated stabilization, specifically of LH enhancing its resistance to clearance. Therefore, in addition to OHSS, our data demonstrate the increased release of oocytes (≥ 15) on COS is associated with low sLHCGR/LH-sLHCGR concentrations (Figure 5 and Table 1). The possibility that an optimal level of sLHCGR complexed with LH exists that promotes pregnancy in those producing 8-14 oocytes might support a physiological reservoir role for sLHCGR in regulating LH availability. In an alternative scenario [27,39,40] the mobile sLHCGR may compete with membrane-associated counterparts for binding to circulating LH/hCG. Therefore, increased levels of serum sLHCGR modulate the LH/hCG activity by inhibiting their interactions with cell surface receptors. In general, high serum sLHCGR concentrations are linked to reduced production of oocytes as well as poor implantation (Figures 5, 6 and Table 1). This could be again linked to the inhibitory effect of circulating sLHCGR on LH/hCG functions.

Pre-treatment serum sLHCGR/LH-sLHCGR and LH levels could provide an indication of functional LH levels that would allow the adjustment of hormone dose prior to ovarian induction. This could be an important step towards avoidance of OHSS, particularly for patients whose AMH levels do not correlate with high oocyte yield and potential OHSS. It should be emphasized that we have measured baseline, pre-treatment sLHCGR concentrations and that during fertility treatment and prior to embryo transfer, these concentrations could alter. Indeed, this might explain both positive and failed implantation when the pre-treatment serum LH receptor concentrations in some women were high and low, respectively (Figure 6). Therefore, the true effect of circulating sLHCGR in modulating implantation and early pregnancy would require its estimation before and immediately after embryo transfer. Future experiments are needed to test this hypothesis.

Critical clinical parameters that predict reduced response to COS are age, BMI, early cycle FSH, LH, inhibin B, antral follicle counts (AFC) and AMH concentrations [5,41]. Of these, serum AMH and AFC are recognized as superior predictors of ovarian reserve and OHSS [8]. Our study indicates that in the absence of AMH data, measurement of pre-treatment circulating sLHCGR/LH-sLHCGR together with FSH:LH ratios, age and AFC might provide sufficient information on aberrant ovarian response to establish individualized hormone combinations, daily doses and duration of treatment prior to COS.

The improvement of pregnancy outcome could be another potential benefit of measuring pre-treatment serum sLHCGR. Following a decline in LH support, hCG is the foremost leutotropic paracrine signal produced by the embryo, well before the implantation begins [7]. This hCG signaling critically influences the blastocyst development, uterine receptivity through stromal fibroblast proliferation, secretion of IGF binding protein-1, NK cell activation and apoptosis (Fas-FasL), transient immune tolerance through activation of regulatory T-cells and dendritic cells (MHC class II, IL-10 and IDO expression), and endometrial angiogenesis through secretion of VEGF [42,43]. Furthermore, an increased LH surge is associated with a high rate of miscarriage [44]. Therefore, measurement of sLHCGR/LH-sLHCGR following embryo transfer together with targeted hCG therapy could improve pregnancy outcome by extending the window of implantation and simultaneously reducing the frequency of miscarriage.

The bioactive serum/plasma LH concentrations, conventionally measured by Leydig cell assay [45] have been shown to be significantly lower than the immunoreactive hormone in various clinical conditions affecting ovarian functions and fertility [46,47]. Whether the ELISA assays described here for measuring the soluble LH receptor and the hormone-receptor complex would be comparable to the Leydig cell assay with respect to LH bioactivity requires a separate investigation. The link between hypogonadism and LHCGR functions was first demonstrated by partial purification of M_r _30 K-60 K serum proteins that were found to competitively inhibit the hormone binding to the LH receptor and affect testosterone production in uremic boys [48].

## Conclusions

Two hormones, LH and hCG use a common cell-surface receptor, known as LHCGR, to transmit signals into cells. Although this receptor is normally tethered to the surface of a cell, new variants (sLHCGR) have been found circulating in the blood. These variants do not transmit signals but may potentiate or inhibit LH and hCG functions. Our group has developed novel assays to measure the level of sLHCGR and LH-sLHCGR complex in women seeking IVF treatment. It was found that those who produced a few oocytes (less than 7) or many oocytes (more than 15), had low concentrations of sLHCGR and a good IVF outcome (i.e. pregnancy), whereas a high level of sLHCGR within these two groups was indicative of a poor IVF outcome (i.e. not pregnant). For those producing an intermediate number of oocytes (8-14) the levels of sLHCGR did not appear to affect pregnancy. These new tests could be useful in avoiding ovarian hyperstimulation and may help circumvent a situation where all embryos need to be frozen. Moreover, if used before uterine transfer of the embryo, these assays may also identify those women who may benefit from short-term supplementation with hCG in order to firmly establish the pregnancy.

## Competing interests

The use of sLHCGR-based immunodiagnostic tests has been patented.

## Authors' contributions

AEC developed the ELISA system, performed all ELISAs, analysed the data, contributed to the interpretation of results, creation of manuscript figures and first draft of the manuscript. KPN collected the patient sera, collated all clinical data and contributed to interpretation of the results. WEM performed statistical analysis on the data and contributed to the creation of manuscript figures. GML presided over the clinical aspect of the study and contributed to the interpretation of results. SB conceived and initiated the study, designed the standards and monoclonal antibodies used in the ELISAs, presided over the statistical analyses, interpretation of results, creation of manuscript figures and the first draft of the manuscript. All authors read and approved the final manuscript.
